# Reviewers

**DOI:** 10.1111/jdi.13141

**Published:** 2019-11-03

The publication of invaluable papers in the Journal of Diabetes Investigation depends on the prompt, careful review of submitted manuscripts. We would like to thank the Editorial Board members, the International Editorial Board members, the Assistant Editorial Board members and the following experts for reviewing manuscripts from September 1, 2018 to August 31, 2019.

Norio Abiru

Ji‐Hyun Ahn

Bo Ahren

Vasudha Ahuja

Toru Aizawa

Hiroshi Akazawa

Mohamed Al‐Shabrawey

Sirajudeen S Alavudeen

Noe Alvarado

Keizo Anzai

Shigeru Aoki

Atsushi Araki

Shin‐ichi Araki

Naoko Arata

Seyed Masoud Arzaghi

Noriko Asahara

Shunichiro Asahara

Yoshimasa Aso

Takuya Awata

Motoharu Awazawa

Masayuki Baba

Sevket Balta

Yuqian Bao

H Berger

H. Bihan

Aldo Bonaventura

Alexandro Bonifaz

Steven Brenner

Magnus Bruze

K. Busiah

Soner Cander

Sven Magnus Carlsen

Ali J. Chakera

Juliana Chan

Dipanjan Chanda

Jer‐Ming Chang

Yi‐cheng Chang

Cheng‐Hsu Chen

Judy Chen

Jui‐Hung Chen

Min Chen

Qingjie Chen

Wei Chen

Ching Lung Cheung

Pik To Cheung

Boon‐How Chew

Anne Chiaramello

SM Ching

Ken Chiu

Sung Hee Choi

Won‐Il Choi

Henrik Christesen

Mark P. Christiansen

NF Chu

Ho Yeon Chung

Rachel Climie

Livia Dainelli

Undurti Das

Buyzere Marc De

Asma Deeb

Takahisa Deguchi

M Dekker

Rick Dobrowsky

Ken Ebihara

Gracia‐Ramos Abraham Edgar

Kazuo Eguchi

Nabil Eid

Gerges Najwa El

Masanori Emoto

Fabio Facchinetti

Gabor Ferneisz

Paul Fernyhough

Ele Ferrannini

Jose L. Flores‐Guerrero

Toshihito Fujii

Rumi Fujikawa

Kei Fujimoto

Shimpei Fujimoto

Tomomi Fujisawa

Yoshihito Fujita

Yukihiro Fujita

Yoshio Fujitani

Maki Fukami

Astunori Fukuhara

Tomoyasu Fukui

Nobuaki Funahashi

Masato Furuhashi

Shinya Furukawa

Hiroto Furuta

Isabel Garcia‐Garcia

Pramit Ghosh

Pieter Gillard

Ali Gol

An Goossens

Atsushi Goto

Katsumasa Goto

Naohiro Gotoh

Haotian Gu

Dingkun Gui

Kyoung Hwa Ha

Yusuke Hada

Kazuyuki Hamaguchi

Tomoya Hamaguchi

Yoshiyuki Hamamoto

Kazuo Hara

Masumi Hara

Shin‐ichi Harashima

Mohamed Azmi Hassali

Yuji Hataya

Yoshitaka Hayashi

Tsutomu Hirano

Takumi Hirata

Takahisa Hirose

Inoue Hiroshi

Yushi Hirota

Mikano Hishiki

Jens Holst

Yukio Horikawa

Masayuki Hosoi

Tetsuya Hosooka

An‐Tsz Hsieh

Chang‐Hsun Hsieh

Hui‐Min Hsieh

Cheng Hu

Gang Hu

Huanhuan Hu

Chia‐Luen Huang

Hannah Hui

Humaira Hussein

Chii‐Min Hwu

Jon Hyett

Yasuo Ido

Katsumi Iizuka

Hiroshi Ikegami

Satoru Ikenoue

Junta Imai

Fumiaki Imamura

Minako Imamura

Toyoshi Inoguchi

Daisuke Inoue

Ikuo Inoue

Mayumi Inoue

Nigel Irwin

Shun Ishibashi

Yasushi Ishigaki

Hisamitsu Ishihara

Hiroshi Ishii

Tatsuya Iso

Hiroto Ito

Hiroshi Itoh

Hiromi Iwahashi

Toshio Iwakura

Naoko Iwasaki

Takanori Iwata

Edward Janus

Mu'taman Jarrar

Chang Hee Jung

Tetsuya Kakuma

Hideki Kamiya

Kyuzi Kamoi

Masahiro Kamouchi

Naotetsu Kanamoto

Keizo Kanasaki

Akio Kanazawa

Ippei Kanazawa

Shizuka Kaneko

Hideaki Kaneto

Yoshifumi Kasuga

Naoto Katakami

Koichi Kato

Yoshiro Kato

Tomoko Kawai

Toshihide Kawai

Takahiko Kawamura

Tomoyuki Kawamura

Daiji Kawanami

Eiji Kawasaki

Jothydev Kesavadev

Mykola Khalangot

Imran Ali Khan

Toru Kikuchi

Chong Hwa Kim

Eun Ky Kim

Kyoung Min Kim

Kyu‐Nam Kim

Mee Kyoung Kim

Minkyung Kim

Nan Hee Kim

Sang Soo Kim

Sang Yong Kim

Tadahiro Kitamura

Arihiro Kiyosue

Tetsuro Kobayashi

Genovefa Kolovou

Mitsuhisa Komatsu

Bokyung Koo

Daisuke Koya

Hidenori Koyama

Peter Kristensen

Akira Kubota

Hiroyuki Kubota

Naoto Kubota

Shinji Kume

Makoto Kurano

Akio Kuroda

Takeshi Kurose

Toru Kusakabe

Hitoshi Kuwata

Sin Man Lam

Gormsen Lars

Lucia Lasala

Ting‐I Lee

Eun Young Lee

Mei‐Yueh Lee

Seung Hwan Lee

Ting‐Wei Lee

Yongho Lee

Yùhuì Lee

Siu‐wai Leung

Hong Li

Hung‐Yuan Li

Jun Li

Qian Li

Xiao‐ying Li

Yuxiu Li

Chia‐Hung Lin

Chia‐Huei Lin

Ming Lin

Buyun Liu

Ming Liu

Giieh‐Hua Lu

Jianhua Ma

Shiro Maeda

Hiroshi Maegawa

Tanmay Mahapatra

Rachid Malek

Rayaz Malik

Alan Maloney

Gomez Ana Maria

Takayuki Masaki

Hisashi Masuyama

Hiroaki Masuzaki

Ikuro Matsuba

Masafumi Matsuda

Munehide Matsuhisa

Michihiro Matsumoto

Taka‐aki Matsuoka

Shu Meguro

Ruilope Luis Miguel

Takashi Miki

Takayuki Miki

Selvaraj Miltonprabu

Takashi Minami

Yasuhiko Minokoshi

Koki Mise

Hirofumi Misu

Tomoya Mita

Junnosuke Miura

Takeshi MIyatsuka

Hideaki Miyoshi

Hiroki Mizukami

Mie Mochizuki

Jun Sung Moon

Thyago Proenca de Moraes

Yasumichi Mori

Jun Morinaga

Katsutaro Morino

Seiichi Munesue

Tomoaki Murakami

Takashi Murata

Alyson Myers

Yoshio Nagai

Shoichiro Nagasaka

Masao Nagata

Jun Nakae

Tomoko Nakagami

Akinobu Nakamura

Mieko Nakamura

Nobuhisa Nakamura

Shuhei Nakanishi

Koji Naruishi

Masami Nemoto

Qin Xiang Ng

Monika Niewczas

Toshiharu Ninomiya

Masahiro Nishi

Tetsuo Nishikawa

Rimei Nishimura

Yoshihiko Nishio

Mitsuhiko Noda

Hiroyuki Nodera

Yoshihiko Noma

Takashi Nomiyama

Shinsuke Noso

Sedigheh Nouhjah

Sumito Ogawa

Yoshihiro Ogawa

Masahito Ogura

Ken Ohashi

Mitsuru Ohsugi

Yasuharu Ohta

Yoichi Oikawa

Yosuke Okada

Yukiko Onishi

Haruhiko Osawa

Tsuguhito Ota

Horng‐Yih Ou

Nobuaki Ozaki

An Pan

Nikolaos Papanas

Kyong Soo Park

Katarzyna Plagens‐Rotman

May Ee Png

Pirkko J Pussinen

Hanbo Qiu

Luis Raposo

Yongcheng Ren

Sang Youl Rhee

Anitha Risberg

Lauren Rodgers

Paola Sacerdote

Yoshifumi Saisho

Kazuhiko Sakaguchi

Juro Sakai

Masaya Sakamoto

Hiroshi Sakaue

Kenichi Sakurai

Jose Luis Sanchez‐Quesada

Sang‐Man Sang‐Man

Kazunori Sango

Motoaki Sano

Hideyuki Sasaki

Toshiyasu Sasaoka

Hiroaki Satoh

Jo Satoh

Yusuke Seino

Dinesh Selvarajah

Hetal Shah

Neel Sharma

YJ Sheen

Kenichi Shikata

Michio Shimabukuro

Akira Shimada

Akira Shimatsu

Yoshihiro Shimazaki

Ikki Shimizu

Masayuki Shimoda

Seiya Shimoda

Jae Yong Shin

Jun Shirakawa

Nobuhiro Shojima

Hirohito Sone

Ki‐Ho Song

Noriyuki Sonoda

Norbert Stefan

Xiaofei Su

Takayoshi Suganami

Takashi Sugiyama

Takashi Sugiyama

Cheuk‐Kwan Sun

Mingzhong Sun

Weiping Sun

Zilin Sun

Atushi Suzuki

Ken Suzuki

Ryou Suzuki

Yasuharu Tabara

Yuji Tajiri

Mitsuyoshi Takahara

Hirokazu Takahashi

Toshinari Takamura

Tetsuo Takehara

Soichi Takeishi

Kyosuke Takeshita

Fumihiko Takeuchi

Yoshikazu Tamori

Kouichi Tamura

Yoshifumi Tamura

Bee Kang Tan

Katsuya Tanabe

Daisuke Tanaka

Nagaaki Tanaka

Tomohiro Tanaka

Jun Tao

Ichiro Tatsuno

Shimosawa Tatsuo

Daisuke Taura

Yasuo Terauchi

Chenggong Tian

Kazuyuki Tobe

Chitoku Toda

Keiichi Torimoto

Satoru Tsujii

Tetsuro Tsujimoto

Shin Tsunekawa

Hiroaki Ueno

Hiroyuki Umegaki

Isao Usui

Kazunori Utsunomiya

Roxana Valdes‐Ramos

Nathalie Viguerie

Jun Wada

Hironori Waki

Chi Chiu Wang

JS Wang

Miao Wang

Yeli Wang

Yufan Wang

Scott White

Jong Chul Won

Connie Woo

Chung‐Ze Wu

Ling Wu

Jianzhong Xiao

Xinhua Xiao

Chihiro Yabe

Kunimasa Yagi

Tetsuya Yamada

Yuichiro Yamada

Kazuya Yamagata

Sho‐ichi Yamagishi

Ritsuko Yamamoto‐Honda

Yasuhiko Yamamoto

Shunsuke Yamane

Masayuki Yamanouchi

Tomoyuki Yamasaki

Yuan‐Horng Yan

Gangyi Yang

T‐Y Yang

Xilin Yang

Zu‐yao Yang

Hisafumi Yasuda

Hitoshi Yasuda

Hiroshi Yatsuya

Rose Yeung

SL Au Yeung

Norihide Yokoi

Hideto Yonekura

Tohru Yorifuji

Dingyun You

Chaohui Yu

Liping Yu

Sueziani Zainudin

Dongyu Zhang

Guangxiang Zhang

Ping Zhang

Xiaolong Zhao

Hongting Zheng

Jian Zhou

Zhiming Zhu

